# Dietary and lifestyle inflammatory scores and risk of incident diabetes: a prospective cohort among participants of Tehran lipid and glucose study

**DOI:** 10.1186/s12889-021-11327-1

**Published:** 2021-07-02

**Authors:** Farshad Teymoori, Hossein Farhadnejad, Ebrahim Mokhtari, Mohammad Hassan Sohouli, Nazanin Moslehi, Parvin Mirmiran, Fereidoun Azizi

**Affiliations:** 1 Nutrition and Endocrine Research Center, Research Institute for Endocrine Sciences, Shahid Beheshti University of Medical Sciences, Tehran, Iran; 2 Department of Nutrition, School of Public Health, Iran University of Medical Sciences, Tehran, Iran; 3 Endocrine Research Center, Research Institute for Endocrine Sciences, Shahid Beheshti University of Medical Sciences, Tehran, Iran

**Keywords:** Diet, Lifestyle, Inflammation, Diabetes

## Abstract

**Background:**

Inflammation is a precursor of chronic disease, which is affected by lifestyle and dietary habits. Recently empirical dietary inflammatory patterns (EDIP), dietary inflammation scores (DIS), and lifestyle inflammation scores (LIS) were developed to indicate lifestyle and dietary contributions in systemic inflammation. The current study aimed to investigate the associations between these indices and the incidence of diabetes among Tehranian adults.

**Methods:**

A total of 4624 individuals, aged 20–75 years, who were free of diabetes at baseline (2008–2011), were followed for 5.71 years (2014–2017) to ascertain incident diabetes. Dietary intakes were collected at baseline using the food frequency questionnaire. The hazard ratio (HR) of diabetes was calculated by Cox proportional hazards regression across quartiles of EDIP, DIS, and LIS, adjusted for potential confounders.

**Results:**

The mean ± SD for the age and BMI of the study population (45.1% male) were 40.8 ± 12.7 years and 27.1 ± 4.1 Kg.m2, respectively. At the end of the follow-up, 329 (7.1%) diabetes cases were identified. In the multivariable-adjusted model, individuals in the highest compared to the lowest quartile of EDIP (HR = 0.83; 95%CI:0.59–1.15, p for trend = 0.286), and LIS (HR = 2.41; 95%CI:1.61–3.60, P for trend < 0.001) had increased risk of diabetes. However, no significant associations were found between the score of DIS and diabetes incidents (HR = 0.83; 95%CI:0.59–1.15, p for trend = 0.286).

**Conclusion:**

Greater adherence to EDIP and LIS scores was associated with a higher risk of diabetes, while no significant association was found between the DIS score and diabetes incident.

**Supplementary Information:**

The online version contains supplementary material available at 10.1186/s12889-021-11327-1.

## Background

Type 2 diabetes mellitus (T2DM) is a severe life-threatening health problem characterized by beta-cells dysfunction, insulin resistance, and high blood glucose levels. This metabolic disease has significant adverse effects on quality of life and increases healthcare costs, comorbidities, and mortality [1]. Through the past recent years, the global prevalence of diabetes has faced a considerable increase and is predicted to rise from approximately 463 million in 2019 to 700 million by 2045 [2].

Chronic inflammation is a well-known risk factor related to chronic diseases, including cardiovascular disease, T2DM, and cancers [3]. Previously, studies have observed significantly higher concentrations of inflammatory factors such as C-reactive protein (CRP), Tumor necrosis factor-α (TNFα), and ILs in individuals with chronic diseases [4, 5]. Also, it has been reported that dietary factors such as high intake of saturated fat and low intake of fruit, vegetables, and whole grains, along with other lifestyle factors including obesity, physical inactivity, and cigarette smoking, collectively play an essential role in the estimation of diet and lifestyle influence on systemic inflammation [6, 7]. So, recently, the researchers sought to determine the inflammation caused by environmental factors such as dietary factors in individuals without directly measuring the serum inflammatory indices and examining its association with the risk of various chronic diseases.

Previously, a pre-defined score called dietary inflammatory index (DII) was developed to assess the contributions of dietary exposures in the body’s inflammatory status and consequently the risk of chronic disease development. In this regard, three cross-sectional [8–10] and prospective [11] studies observed a higher DII score positively associated with a higher risk of diabetes. DII estimates the potential relation of selected food parameters with the body’s inflammatory status [12]. DII mostly focuses on the anti/pro-inflammatory nutrients without considering the nutrient interactions in body homeostasis and other effects of unmeasured and unknown anti/pro-inflammatory compounds of whole foods and beverages. Recently two novels inflammatory indices, including empirical dietary inflammatory pattern (EDIP) and dietary inflammation scores (DIS), have been proposed based on the association between food groups and inflammatory markers [13, 14].

Furthermore, lifestyle inflammation scores (LIS) have been introduced to address lifestyle characteristics’ cumulative contributions, including body mass index, physical activity, alcohol consumption, and smoking to body inflammation status [13, 14]. Evidence about EDIP is still limited. Recently, two studies have suggested a positive link between EDIP and diabetes [15, 16]. Furthermore, the relationship between this index and other outcomes has been somewhat inconsistent [17–20]. To our knowledge, no study has yet investigated the relationship between DIS, LIS with risk of T2DM. Some previous studies have been assessed the association of these two novel inflammation scores with the risk of chronic diseases such as metabolic syndrome, cancers, inflammatory bowel disease (IBD), and CVD risk factors [21–24].

The current study is aimed to assess the associations of DIS, LIS, and EDIP with the incidence of T2DM among adult participants in a population-based study, Tehran Lipid and Glucose Study (TLGS).

## Method

### Study participants

The present study was conducted within the framework of the Tehran Lipid and Glucose Study (TLGS), which was conducted to determine the risk factors for non-communicable diseases among a representative urban population of Tehran, including 15,005 participants aged ≥3 years. The TLGS is an ongoing population-based prospective study initiated in 1999 (First phase), and its data are being collected prospectively at 3 y intervals. Written informed consent was obtained from all participants. All methods were carried out in accordance with relevant guidelines and regulations. Details of the TLGS have been reported previously [25].

**Fig. 1:**
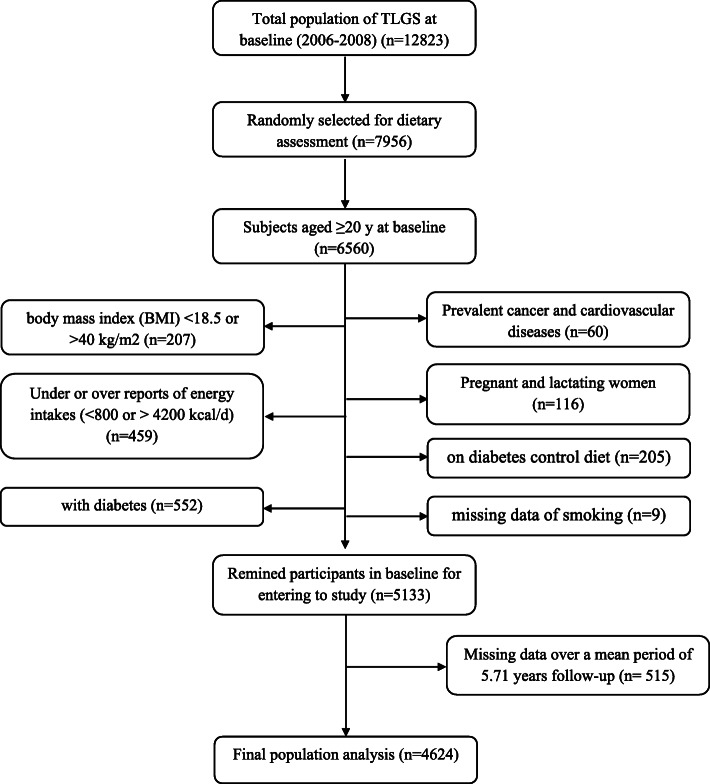
Flow-diagram of the Tehran Lipid and Glucose study population

In the fourth phase of the TLGS (2009–2011), 12,823 participants, 7956 randomly selected, agreed to complete the dietary assessment. For the current study, a total of 6560 individuals, aged > 20 years old, with complete dietary data in the fourth examination of TLGS, as a baseline examination, were enrolled. Participants with under-reporting or over-reporting dietary intakes (energy intakes of less than 800 kcal/d or more than 4500 kcal/d, respectively; these values between the ±3 SD of mean energy intake in the study population), (*n* = 459), those on diabetes control diet (*n* = 205), participants with diabetes (*n* = 552), those with a history of myocardial infarction, cerebral vascular accident, cancers (*n* = 60), individuals with body mass index (BMI) < 18.5 or > 40 kg/m^2^ (*n* = 207), lactating and pregnant women (*n* = 116), and those with missing data of smoking (*n* = 9) were excluded in the baseline of the study. Some individuals fell into more than one exclusion category. From 5139 participants who were followed up until the sixth phase of TLGS (2015–18), over a mean period of 5.71 years, 515 participants were missed to follow up, and 4624 subjects remained for the final analyses (follow up rate = 90%) (Fig. 1).

### Dietary intake assessment

Dietary intakes at baseline were assessed using a valid and reliable semi-quantitative food frequency questionnaire (FFQ) [26]. The reliability and validity of the FFQ have been previously reported. During the previous year, the consumption frequency of each food item daily, weekly, or monthly was collected during a face-to-face interview by trained and experienced dieticians. Portion sizes of consumed foods reported in household measures were then converted into grams. Using the United States Department of Agriculture (USDA) food composition table (FCT), energy and nutrient contents were computed. The Iranian FCT was used for local food items that were not available in USDA FCT.

### Physical activity assessment

The modifiable activity questionnaire (MAQ) was used for assessing physical activity levels in participants. This questionnaire has previously been modified and validated among the Iranian population [27]; individuals were asked to report and identify the frequency and time spent on activities of light, moderate, hard, and very hard intensity, during the past 12 months, according to a list of everyday activities of daily life; physical activity levels were expressed as metabolic equivalent hours per week.

### Clinical and biochemical measurements

Information on age, sex, medical history, medication use, and participants’ smoking habits were collected by trained interviewers using pretested questionnaires. The subjects’ body weight was measured to the nearest 100 g using digital scales (model 707, Seca, Hamburg, Germany) while lightly clothed and not wearing shoes. Height was measured to the nearest 0.5 cm with a tape measure (model 208 Portable Body Meter Measuring Device; Seca) while the subjects were barefoot in the standing position. Body mass index (BMI) was computed as weight in kilograms, divided by height in meters squared. Waist circumference (WC) was measured to the nearest 0.1 cm using an un-stretched tape meter, at the umbilicus level, over light clothing, without any pressure on the body surface.

BP was measured twice with a minimum interval of 30 s from the right arm after resting for at least 15 min while sitting on a chair, using a mercury sphygmomanometer and the Korotkoff sound technique with an accuracy of 2 mmHg; the mean of the two measurements was regarded as the participant’s blood pressure.

Blood samples were taken and transferred into vacutainer tubes between 7:00 and 9:00 a.m., after a 12–14-h overnight fast, while subjects were in a sitting position. Blood samples were centrifuged within 30 to 45 min of collection. All biochemical analyses were performed using a Selectra 2 auto-analyzer at the TLGS research laboratory on the day of blood collection. Fasting blood sugar (FBS) was measured using an enzymatic colorimetric method with glucose oxidase. Inter- and intra-assay CVs were both 2.2% for FBS [25]. Triglyceride (TGs) levels were measured using the enzymatic colorimetric method with glycerol phosphate oxidase. Inter- and intra-assay CVs for TGs were 0.6 and 1.6%, respectively. Serum high-density lipoprotein-cholesterol (HDL-C) was measured after precipitation of the apolipoprotein B-containing lipoproteins with phosphotungstic acid. Enzymatic colorimetric tests were used to assay total cholesterol (TC) with cholesterol esterase and cholesterol oxidase. Inter- and intra-assay CVs for both TC and HDL-C were 0.5 and 2%, respectively. Friedewald formula was used to calculate low-density lipoprotein-cholesterol (LDL-C) expressed in mg/dL. Biochemical measurements were performed using commercial kits (Pars Azmoon Inc., Tehran, Iran).

### Calculation of indices

Dietary data derived from FFQ were used to calculate inflammatory scores. The dietary (DIS) and lifestyle (LIS) inflammation scores were recently proposed by Byrd D.A et al. [13]. DIS includes 19 component initially, but because we have no information about supplement intake, we calculated the overall score with 18 food groups, including leafy greens and cruciferous vegetables, tomatoes, apples and berries, deep yellow or orange vegetables and fruit, other fruits, and real fruit juices, other vegetables, legumes, fish, poultry, red and organ meats, processed meats, added sugars, high-fat dairy, low-fat dairy and tea, nuts, other fats, refined grains, and starchy vegetables. We standardized each food group (to a mean of zero and SD of 1), and then the values were summed [13] (Table S1).

Physical activity, BMI, and smoking status were used to calculate the LIS score. Due to religious and legal restrictions in the Iranian population, alcohol is not consumed, or its consumption is not reported, so we ignored alcohol consumption to calculate LIS. First, a dummy variable was created from each component and then multiplied for proposed regression coefficients as follows [13] (Table S1).

Physical activity was categorized into tertiles, and participants in the first, second, and third tertiles give the score of 0.0, − 0.18, and − 0.41, respectively. Participants were categorized into average weight (BMI < 25), overweight (25 ≤ BMI < 30), and obese (BMI ≥ 30); and then respectively received 0.0. 0.89 and 1.57 scores. Also, the proposed regression coefficients for smokers vs. non-smokers were 0.50 vs. 0.0, which were assigned. Finally, all the weighted values were summed to calculate the LIS score.

The EDIP scores were calculated based on the Tabung et al. study [14]. Since alcoholic drinks such as wine and beer are not common or maybe unreported in the Iranian population due to religious considerations, we do not include them in calculating indices. Because we have no food items as low-energy beverages in our FFQ, the food item was also excluded. Therefore we calculated EDIP score with 15 instead of 18 food group based on dietary intakes of processed meat (sausage), red meat (beef, or lamb), organ meat (beef, calf, or chicken liver), other fish (canned tuna, or fish), other vegetables (mixed vegetables, green pepper, cooked mushroom, eggplant, zucchini, or cucumber), refined grains (white bread, biscuit, white rice, pasta, or vermicelli), high-energy and low energy beverages (cola with sugar, carbonated beverages with sugar, fruit punch drinks), and tomatoes as pro-inflammatory group and tea, coffee, dark yellow vegetables (carrots, or squash), leafy green vegetables (cabbage, spinach, or lettuce), snacks (cracker, or potato chips), fruit juice (apple juice, cantaloupe juice, orange juice, or other fruit juice), pizza as an anti-inflammatory group. The mean daily intakes of each food group were multiplied by the proposed regression coefficients, and then all the weighted values were summed [14] (Table S1). Finally, the summed scores were divided by 1000 to reduce the magnitude of the scores. In every three indexes, a higher score indicates a more pro-inflammatory diet and vice versa.

### Definitions of terms

T2DM was defined based on the American Diabetes Association’s criteria as having FBS levels ≥126 mg/dl or 2-h post-75 g glucose loads ≥200 mg/dl or treating with hypoglycaemic drugs [28]. Also, for the definition of hypertension, subjects who had systolic blood pressure levels higher than 140 or diastolic blood pressure levels of 90 mmHg or consumed antihypertensive medications were considered hypertensive [29].

### Statistical analysis

Data analyses were conducted using the Statistical Package for Social Sciences (version 20.0; SPSS Inc., Chicago, IL). The normality of variables was assessed using histogram charts and Kolmogorov–Smirnov analysis. Baseline characteristics of subjects were expressed as mean ± SD or median (25–75) interquartile range (IQR) for continuous variables and percentage for categorical variables, respectively. An independent sample t-test and chi-square were used for comparing the quantitative and qualitative variables between diabetic and healthy participants.

Individuals’ duration of follow-up (in the year) were calculated from baseline to the time at which an event (definitive diagnosis of diabetes by endocrinologist based on the criteria as mentioned above) occurred for the first time (event date) or the last date of follow up examination, whichever occurred first. The event date of occurrence of the diabetes was determined as mid-time between the date of the follow-up visit at which the diabetes was detected for the first time and the most recent follow-up visit preceding the diagnosis.

Subjects were categorized according to quartiles of EDIP, DIS, and LIS. Cox proportional hazard regression used to estimate the hazard ratio and 95% confidence intervals (HRs and 95% CIs) of diabetes incident across quartiles of the EDIP, DIS, and LIS scores adjusted for potential confounders including age, sex, BMI, physical activity, smoking, daily energy intake, education level, hypertension, fasting blood sugar, and TG to HDL ratio.

We conducted a sensitivity analysis by excluding the smokers, participants with a family history of diabetes, and hypertensive patients; then, we repeated the analysis of the association between inflammatory factors with diabetes incidents. As smoking was a component of the LIS score, by excluding the smokers, LIS was unpredictable, so sensitivity analysis among the non-smoker’s population was just conducted for assessing the association of EDIP and DIS with diabetes incidence.

We also stratified our data based on the BMI (BMI < 25, BMI ≥ 25) and physical activity categories (lower than the median, higher than the median) and analyzed the association between EDIP and DIS with diabetes incidents adjusted for the variables as mentioned above across these categories. *P*-values < .05 were considered as statistically significant.

## Result

The mean ± SD for the age and BMI of the study population were 40.8 ± 12.7 years and 27.1 ± 4.1 Kg.m2, respectively, and 45.1% of participants were male. Also, the median (IQR) of EDIP, LIS, and DIS were 0.47 (0.30, 0.70), 0.71(0.00, 1.16), and 0.06(− 0.51, 0.61), respectively. During an average of 5.71 years of follow-up, 329 (7.1%) new cases of diabetes were ascertained.

The Baseline characteristics and dietary intakes of patients are presented in Table 1. Compared with non-diabetic subjects, participants with diabetes were significantly older and had higher BMI, FBS, TGs, SBP, DBP, and TG: HDL ratio, a higher percentage of hypertension, and lower HDL-C academic education level.

**Table 1:** Baseline characteristics of participants according to the development of diabetes during the follow-up

	Diabetes (***n*** = 329)	Healthy (***n*** = 4295)	*P*-value
Age(year)	47.7 (12.4)	40.3 (12.6)	< 0.001
Male, n (%)	158 (48.0)	1928 (44.9)	0.271
Body mass index (Kg/m^2^)	29.7 (4.1)	27.0 (4.1)	< 0.001
Physical Activity (Met.min/wk)	73.3 (53.6)	74.5 (54.2)	0.356
Academic education (graduated), n (%)	65 (19.8)	1297 (30.2)	< 0.001
Employed, n (%)	272 (82.7)	3611 (84.1)	0.411
Smoking, n (%)	40 (12.2)	482 (11.2)	0.609
**Biochemical data**			
Fasting blood sugar (mg/dl)	101.9 (11.1)	92.0 (7.8)	< 0.001
Triglycerides (mg/dl)	189.1 (95.6)	133.2 (80.6)	< 0.001
High density lipoprotein- Cholesterol (mg/dl)	44.4 (11.0)	47.8 (11.4)	< 0.001
TG:HDL ratio	4.7 (3.1)	3.1 (2.6)	< 0.001
Systolic blood pressure (mmHg)	122.1 (17.9)	111.8 (14.8)	< 0.001
Diastolic blood pressure (mmHg)	79.6 (12.2)	75.2 (10.3)	< 0.001
Hypertension (%)	107 (32.5)	583 (13.6)	< 0.001

Data are presented as mean (SD) for continuous variable and number (percent) for categorical variables

*TG:HDL ratio* Triglycerides: High density lipoprotein- Cholesterol ratio

The components of three inflammatory indices, including EDIP, LIS, and DIS among diabetic and healthy participants, are shown in Table 2. The intakes of energy, macronutrients were not significantly different between the two groups. In comparison to non-diabetic patients, diabetic subjects had a healthier diet based on the DIS because they have a significantly lower score of DIS and also higher “other vegetables” consumption as a DIS component (*P*-value = 0.05). Compared with non-diabetic subjects, participants with diabetes had higher EDIP scores, and among EDIP components, “other vegetables” consumption, however, had a lower intake of snacks (*P*-value = 0.05). LIS scores and percentage of obesity or overweight (higher BMI) as a component of LIS were higher among diabetic patients (*P*-value = 0.05).

**Table 2:** Dietary EDIP, DIS and LIS components intakes of participants’ according to their status of type 2 diabetes: Tehran Lipid and Glucose study

	Diabetes (*n* = 329)	Healthy (*n* = 4295)	*P*-value
**Nutrients**			
Energy (Kcal/d)	2407 (704)	2428 (747)	0.593
Carbohydrate (% of energy)	58.9 (7.1)	59.0 (8.5)	0.845
Protein (% of energy)	15.0 (2.6)	15.0 (6.9)	0.949
Fat (% of energy)	29.6 (6.2)	30.0 (14.6)	0.310
**EDIP component**			
**EDIP score**	0.59 (0.44)	0.53 (0.39)	0.032
Processed meat(serving/d)	0.02 (0.00–0.04)	0.02 (0.00–0.04)	0.611
Red meat(serving/d)	0.09 (0.06–0.16)	0.09 (0.05–0.16)	0.419
Organ meat(serving/d)	0.01 (0.00–0.02)	0.01 (0.00–0.02)	0.464
Other fish(serving/d)	0.06 (0.03–0.13)	0.06 (0.03–0.13)	0.841
Other vegetables(serving/d)	2.67 (2.28)	2.38 (1.91)	0.024
Refined grains(serving/d)	3.52 (2.92)	3.34 (2.49)	0.303
High-energy beverages(serving/d)	0.05 (0.01–0.13)	0.05 (0.01–0.11)	0.713
Tomatoes(serving/d)	0.64 (0.32–1.12)	0.64 (0.32–1.12)	0.138
Tea(serving/d)	2.56 (2.09)	2.43 (2.07)	0.241
Coffee(serving/d)	0.00 (0.00–0.02)	0.00 (0.00–0.02)	0.998
Dark yellow vegetables(serving/d)	0.13 (0.05–0.29)	0.13 (0.05–0.29)	0.609
Leafy green vegetables(serving/d)	0.43 (0.21–0.77)	0.37 (0.18–0.68)	0.174
Snacks(serving/d)	0.13 (0.01–0.36)	0.15 (0.03–0.41)	0.019
Fruit juice(serving/d)	0.04 (0.01–0.11)	0.04 (0.01–0.12)	0.771
Pizza(serving/d)	0.01 (0.00–0.03)	0.01 (0.00–0.03)	0.852
**DIS component**			
**DIS score**	−0.07 (−3.36, 2.89)	0.07 (−0.50–0.62)	0.006
Leafy greens and Cruciferous vegetables (g/d)	23.4 (11.1–41.6)	20.7 (10.2–37.5)	0.263
Tomatoes(g/d)	81.0 (45.6–173.8)	79.0 (41.8–137.8)	0.157
Apples and berries(g/d)	68.8 (35.7–131.3)	64.2 (27.3–126.7)	0.461
Deep yellow or orange Vegetables and fruit(g/d)	40.5 (20.0–79.9)	37.2 (19.4–74.1)	0.786
Other fruits and real fruit juices(g/d)	233 (128–390)	222 (116–383)	0.333
Other vegetables(g/d)	187 (128)	163 (108)	0.001
Legumes(g/d)	47.9 (43.8)	46.2 (42.2)	0.503
Fish(g/d)	6.4 (3.3–14.5)	6.7 (3.6–14.6)	0.841
Poultry(g/d)	24.3 (12.1–36.4)	24.3 (12.1–36.4)	0.318
Red and organ meats(g/d)	33.9 (23.4)	36.1 (27.1)	0.103
Processed meats(g/d)	2.66 (0.49–5.33)	2.41 (0.49–5.33)	0.611
Added sugars(g/d)	57.0 (28.8–120.9)	59.8 (31.4–117.1)	0.920
High-fat dairy(g/d)	82.9 (20.4–234.6)	99.9 (30.3–233.9)	0.418
Low-fat dairy(g/d)	193 (166)	181 (151)	0.209
Coffee and tea(g/d)	628 (507)	595 (499)	0.246
Nuts(g/d)	4.59 (2.07–10.41)	4.40 (2.06–9.11)	0.133
Other fats(g/d)	24.5 (18.8)	25.7 (19.4)	0.274
Refined grains and Starchy vegetables(g/d)	477 (205)	494 (218)	0.151
**LIS Component**			
**LIS score**	0.98 (0.71–1.39)	0.71 (0.00–0.98)	< 0.001
Current smoker	40 (12.2)	428 (11.2)	0.609
Physical activity categories			0.787
Moderately physically active	108 (32.8)	1428 (33.2)	
Heavily physically active	108 (32.8)	1428 (33.2)	
*BMI categories*			< 0.001
Overweight (BMI = 25–29.9)	138 (41.9)	1928 (44.9)	
Obese (BMI ≥30)	150 (45.6)	937 (21.8)	

Data are presented as mean (standard deviation) for normally distributed variables and median (25–75 interquartile range) for skewed variables

*EDIP* empirical dietary inflammatory pattern, *DIS* dietary inflammation scores, *LIS* lifestyle inflammation scores

The association of EDIP, DIS, and LIS with the risk of incident diabetes is shown in Table 3. In the age and sex-adjusted model, the odds of diabetes were higher in individuals in the highest quartiles of the EDIP (HR = 1.60; 95%CI:1.17–2.19, p for trend = 0.009) and LIS (HR = 4.26; 95%CI:2.88–6.30, p for trend< 0.001) compared to those in the lowest quartile of these scores; However, there was no statically significant association between DIS and risk of diabetes (HR = 0.83; 95%CI:0.59–1.15, p for trend = 0.286). In the final model (model 3), after further adjustment for BMI, smoking, physical activity (only for EDIP and DIS), energy, education level, hypertension, fasting blood sugar, and TG to HDL ratio, participants in the highest vs. lowest quartile of LIS (HR = 2.41; 95%CI:1.61–3.60, P for trend < 0.001), and EDIP (HR = 1.52; 95%CI:0. 1.08–2.14, P for trend = 0. 0.038) has a higher risk of diabetes incident. However, according to each of the three models, a higher score of DIS showed no significant association with the risk of diabetes.

**Table 3:** Hazard ratio (95% confidence intervals) of developing diabetes based on quartiles of inflammatory indices

	Quartiles of scores				P for trend
	Q1	Q2	Q3	Q4	
**EDIP**					
Median score	0.19	0.39	0.57	0.91	
Case/Total	71 / 1156	90 / 1154	74 / 1156	94 / 1155	
Follow-up time	5.76 (1.51)	5.69 (1.55)	5.74 (1.57)	5.66 (1.640	
Model 1*	1.00 (Ref)	1.36 (0.99–1.88)	1.16 (0.83–1.63)	1.60 (1.17–2.19)	0.009
Model 2^†^	1.00 (Ref)	1.36 (0.99–1.88)	1.11 (0.79–1.58)	1.55 (1.11–2.17)	0.026
Model 3^‡^	1.00 (Ref)	1.40 (1.01–1.93)	1.32 (0.86–1.72)	1.52 (1.08–2.14)	0.038
**DIS**					
Follow-up time	5.71 (1.58)	5.71 (1.57)	5.69 (1.58)	5.74 (1.55)	
Median score	−1.01	−0.21	0.32	1.01	
Case/Total	100 / 1155	87 / 1155	80 / 1155	62 / 1155	
Model 1*	1.00 (Ref)	0.96 (0.72–1.30)	0.95 (0.70–1.28)	0.83 (0.59–1.15)	0.286
Model 2^†^	1.00 (Ref)	1.06 (0.79–1.44)	1.02 (0.75–1.40)	0.91 (0.65–1.28)	0.640
Model 3^‡^	1.00 (Ref)	0.85 (0.72–1.31)	0.89 (0.71–1.34)	0.36 (0.61–1.20)	0.418
**LIS**					
Follow-up time	5.80 (1.48)	5.80 (1.51)	5.73 (1.54)	5.51 (1.72)	
Median score	−0.18	0.48	0.89	1.39	
Case/Total	34 / 1297	84 / 1401	53 / 721	157 / 1201	
Model 1*	1.00 (Ref)	1.99 (1.31–3.01)	2.29 (1.46–3.59)	4.26 (2.88–6.30)	< 0.001
Model 2^¶^	1.00 (Ref)	1.97 (1.30–2.99)	2.26 (1.44–3.56)	4.18 (2.83–6.20)	< 0.001
Model 3^§^	1.00 (Ref)	1.53 (1.01–2.34)	1.38 (0.87–2.21)	2.41 (1.61–3.60)	< 0.001

*Model 1: adjusted for age and sex.

^†^Model 2: adjusted for model 1 and energy, body mass index, smoking, physical activity, education level

^‡^Model 3: adjusted for model 2 and hypertension, fasting blood sugar, and TG to HDL ratio

^¶^Model2: adjusted for model 1 and energy and education level

^§^Model 3: adjusted for model 1 and energy, education level, hypertension, fasting blood sugar, and TG to HDL ratio

Table 4 presents the association of inflammatory indices and the risk of diabetes incident among the participants who were on the highest compared with lowest quartiles of EDIP, DIS, and LIS using a sensitivity analysis based on the excluding of smokers, participants with family history of diabetes, and hypertensive patients.

**Table 4:** Hazard ratio (95% confidence intervals) of developing diabetes based on quartiles of inflammatory indices after excluding the smokers, participants with family history of diabetes, and Hypertensive patients

	Quartiles of scores				P for trend
	Q1	Q2	Q3	Q4	
**Smoker excluded (*N*** **= 4102)**					
EDIP	1.00 (ref)	1.47 (1.03–2.08)	1.30 (0.89–1.88)	1.65 (1.15–2.38)	0.018
DIS	1.00 (ref)	1.02 (0.74–1.41)	1.00 (0.72–1.40)	0.88 (0.61–1.26)	0.522
**participants with family history of diabetes excluded (*N*** **= 4152)**^**‡**^					
EDIP	1.00 (ref)	1.56 (1.10–2.21)	1.30 (0.90–1.89)	1.55 (1.07–2.25)	0.064
DIS	1.00 (ref)	1.00 (0.72–1.39)	0.99 (0.71–1.39)	0.93 (0.65–1.33)	0.717
LIS	1.00 (ref)	1.50 (0.97–2.31)	1.32 (0.80–2.16)	2.20 (1.44–3.36)	< 0.001
**Hypertensive patients excluded (*N*** **= 3926)**^**§**^					
EDIP	1.00 (ref)	1.40 (0.94–2.07)	1.31 (0.86–2.00)	1.59 (1.05–2.40)	0.049
DIS	1.00 (ref)	1.01 (0.71–1.46)	1.11 (0.76–1.61)	0.80 (0.53–1.21)	0.448
LIS	1.00 (ref)	1.58 (0.98–2.54)	1.65 (0.98–2.78)	2.41 (1.51–3.83)	< 0.001

^‡^adjusted for age, sex, energy, body mass index, smoking, physical activity, education level, hypertension, fasting blood sugar, and TG to HDL ratio

^§^adjusted for age, sex, energy, education level, hypertension, fasting blood sugar, and TG to HDL ratio

In the final adjusted models for potential variables, the highest vs. lowest quartile of EDIP significantly associated with a higher risk of T2DM (HR: 1.65, 95% CI 1.15–2.38, P for trend:0.018), (HR: 1.55, 95% CI 1.07–2.25, P for trend:0.064), (HR: 1.59, 95% CI 1.05–2.40, P for trend:0.049) after excluding of smokers, participants with family history of diabetes, and hypertensive patients, respectively. However, no significant association was observed between a higher score of DIS and the risk of T2DM.

Also, the direct association between LIS and diabetes incident was repeated in sensitivity analysis; as the HR and 95%CI of diabetes incidents among participants in the highest compared to lowest quartiles of LIS were 2.20 (1.44–3.36), P for trend < 0.001 and 2.41 (1.51–3.83), P for trend < 0.001 after excluding the participants with family history of diabetes and hypertensive patients, respectively.

**Fig. 2:**
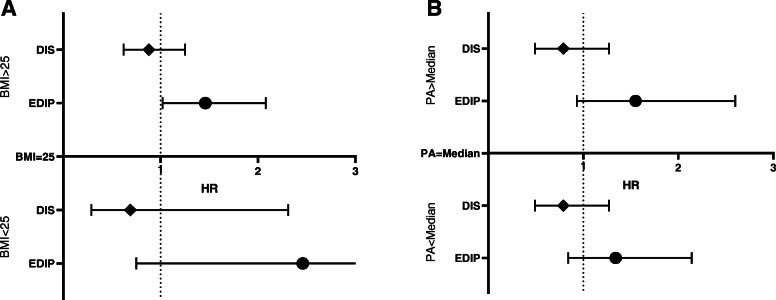
**(A-B):** Stratified analysis for the risk of type 2 diabetes among participants in the highest vs. lowest quartile of DIS and EDIP based on the categories of body mass index (A) and physical activity (B)

Stratified analyses were performed based on the BMI (BMI < 25, BMI ≥ 25) and physical activity classification (lower than the median, higher than the median) to assess the association of DIS and EDIP with risk of diabetes incidents across these categories, and findings were reported in Fig. 2. Based on the stratified analysis, in participants with a BMI ≥ of 25, after adjusting for variables as mentioned earlier, a higher score of EDIP significantly associated with a higher risk of T2DM (HR: 1.46, 95% CI 1.02–2.08, P for trend:0.070) (Fig. 2A). However, no significant association was observed between a higher score of EDIP and the risk of T2DM in subjects with BMI < 25. Also, there is no significant association between the higher score of DIS risk of T2DM in participants based on BMI classification (BMI < 25, BMI ≥ 25) (Fig. 2A). Furthermore, stratified analysis according to physical activity categories (lower than the median, higher than the median) showed no significant relationship between DIS and EDIP score with risk of T2DM (Fig. 2B).

## Discussion

In the present study, we used three indices, including EDIP, DIS, and LIS, to evaluate the association between the inflammatory potential of diet and lifestyle with the risk of diabetes among Tehranian adults. Our findings suggest that greater adherence to a pro-inflammatory diet with higher scores of EDIP associated with a higher risk of diabetes, while no significant association was found between DIS score and the risk of diabetes; also, we found a positive association between LIS score, as an indicator of lifestyle influences on inflammatory status, and incidence of diabetes after 5.71 years follow-up. Also, sensitivity analyses based on BMI and physical activity classification somewhat showed repeated results on the association of DIS and EDIP with risk of T2DM. However, stratified analysis based on BMI categorization suggested that a higher score of EDIP represents a pro-inflammatory dietary pattern, increases the risk of developing T2DM if individuals have an excessive BMI.

To the best of our knowledge, the association between DIS and LIS and the risk of diabetes has not been investigated previously. However, recently two studies have been conducted on the relationship of EDIP with diabetes risk [15, 16]. Also, other epidemiological studies have assessed the association between these inflammatory indices and the risk of various chronic diseases such as metabolic syndrome, CVD risk factors, cancers, and IBD, where the reported findings are controversial [19, 20, 30–32]. Shakeri, Z et al. investigated the relationship between EDIP and MetS and its component and found that a pro-inflammatory diet is a risk factor for the development of MetS, hyperglycemia, low HDL-C, and central obesity [19]. Also, Soltani et al., In a cross-sectional study, observed that overweight/obese individuals with higher EDIP scores have an increased odds of unhealthy metabolic phenotype, high FBS, low-HDL-C, and lower waist circumference [20]. These two cross-sectional studies showed that EDIP is associated with greater odds of diabetes risk factors. Two prospective studies conducted by Byrd et al. found that the inflammatory potential of diet, measured by DIS and LIS [33, 34], may increase the risk of all-cause, cancer- and CVD-specific mortality [33] and colorectal cancer [34].

Furthermore, two prospective studies showed that a higher EDIP score could increase the risk of colorectal cancer [35]; however, other investigations on ovarian cancer [32] and bladder cancer [30] was found no association. Our findings on the association between EDIP and LIS score with the risk of diabetes incidence are consistent with the results of three cross-sectional studies that observed a higher DII score positively associated with a higher risk of diabetes [8–10]. Also, in a cohort study by Laouali et al., an adapted dietary inflammatory index (ADII) showed that greater adherence to an anti-inflammatory diet is associated with a lower risk of T2DM [11].

Our findings showed a 56% higher risk of diabetes among participants in the highest quartile of EDIP than those in the lowest quartile. These results are in line with two recently published prospective studies [15, 16]. Lee et al. demonstrated that individuals who consumed a pro-inflammatory diet (determined by higher EDIP score) are at three-time higher risk of diabetes [16]. Another study among postmenopausal women suggests that participants in the highest quintile of EDIP score have a 0.45% greater risk of diabetes; they also found that reducing the inflammatory potentials of the diet may have more preventive effects against diabetes than focusing only on glycemic foods [15]. EDIP, as a data-driven index, has focused mainly on that part of the diet, which seems to have more positive correlations with inflammatory markers, as we found positive values of EDIP for most participants (about 97%), which shows this score highlights pro-inflammatory aspects of diet. The impact of the major anti-inflammatory contributors of the score was attenuated in our study for some reason; consumption of alcoholic beverages was unreported in our data due to religious considerations, and also coffee and pizza intake were too low. So, if these limitations did not exist, the individuals might be ranked in a broader range of scores, and it provides a better condition for comparing participants in different levels of EDIP.

In the present study, unlike the EDIP index, the DIS showed no association with the risk of T2DM. This finding mostly resulted from nutritional behavior differences between diabetic and non-diabetic participants as we observed a relatively healthier diet among diabetic participants. Besides, DIS is a dietary inflammatory index based on food groups that focus on a wide range of dietary foods; however, it provides a more realistic assessment of usual dietary intake. The interaction of anti/pro-inflammatory foods can attenuate its estimation abilities for disease risk. Moreover, this index has been developed and validated for the US population, and the given weights have been calculated based on their dietary patterns and eating habits, so further studies are needed to assess its applicability in our population; as in our study, unexpectedly non-diabetic participants significantly had a higher DIS score. In addition, based on our findings, the LIS score was more robust than the DIS and EDIP in the estimation of the increment T2DM incident; This substantial increase in the risk of T2DM was seen especially in the fourth quartile of LIS; based on baseline findings, individuals in the fourth quartile of LIS had an unfavorable condition in terms of the lifestyle components, including physical activity level (36% low active), obesity (90% obese) and smoking (19% smoked). Each of these environmental factors can be as important as the dietary pattern in increasing inflammatory conditions and predisposing individuals to chronic diseases such as T2DM. This claim and hypothesis can be confirmed by results of stratified analysis on the association of EDIP with risk of T2DM, which showed that when an unfavorable environmental factor such as obesity is combined with an improper diet, the inflammatory condition can be exacerbated and predispose individuals to T2DM. Therefore, it was expected that the cooperative contributions of major lifestyle-related determinants such as BMI, physical activity, and smoking to inflammation, as a single inflammatory index (LIS), showed a stronger relationship with increased risk of T2DM than the DIS as an alone dietary inflammatory score.

LIS, which addresses cooperative contributions of main lifestyle-related factors, including BMI, physical activity, and smoking to inflammation, showed a more substantial relationship with diabetes than DIS and EDIP. It seems the combination of the LIS components at the continuous form with dietary pro/anti-inflammatory foods in one index can considerably improve the ability to estimate the risk of diabetes. Lifestyle-related factors such as BMI, physical activity, and smoking can significantly influence the inflammatory status and insulin homeostasis. High BMI and increased adipose tissue are positively associated with inflammatory markers such as TNF-α, insulin resistance, and β-cell dysfunction [6, 10, 36–38]. Furthermore, smoking has been proposed as an independent risk factor for diabetes via its detrimental impact on β-cells dysfunction and IR, mostly related to excessive production of harmful tissues, up-regulating inflammatory biomarkers, and cytokines [39–41]. Also, cigarette nicotine exposure significantly decreases insulin sensitivity through p44/p42 MAPK and mTOR pathways [40]. Moderate to high physical activity is another LIS component that has protective effects on chronic inflammation via its ability to improves plasma antioxidant capacity, inducing anti-inflammatory cytokines production, reducing vascular wall inflammation [42, 43], desirable alteration in the lipid-deposition pattern and lowering body fat mass through negative energy balance [44].

This study had several strengths; it was a relatively large population-based prospective study with long-term follow-up, which firstly assessed the association of LIS and DIS with the risk of diabetes incidence globally and EDIP in an Asian population. We also used valid and reliable food-frequency and physical activity questionnaires for dietary and physical activity and filled them with expert interviewers during a face-to-face interview. Despite these strengths, some limitations of the present study should also be mentioned. Firstly, 15 out of 18 food groups were used to calculate the EDIP score in this study. Some items were also excluded in the calculation of DIS and LIS. The final scores were computed based on 18 instead of 19 for DIS and 3 instead of 4 for LIS. Secondly, similar to all nutritional studies using FFQ, measurement error is a potential concern. Thirdly, there is possible residual confounding that we cannot exclude due to unknown or unmeasured factors. Another possible limitation is that these indices have been validated for the US population, and using the given weight may not be favorably applicable in other populations. However, the lack of validated and reliable inflammatory biomarker panel and similar previous studies with a different population lead us to use these indices in our study.

## Conclusions

This prospective, population-based study showed that higher EDIP and LIS scores were associated with an increased risk of diabetes in adults; however, DIS showed no significant association with diabetes incidence.

Further epidemiological studies are needed to address the role of the inflammatory potential of diet and lifestyle and their combinations in the risk of diabetes and its potential mechanisms.

## Supplementary Information

**Additional file 1: Table S1.** Components of the EDIP, DIS, and LIS indices.

## Data Availability

The datasets analysed in the current study are available from the corresponding author on reasonable request.

## Abbreviations

T2DM: Type 2 diabetes mellitus
CVD: Cardiovascular disease
EDIP: Empirical dietary inflammatory pattern
DIS: Dietary inflammation scores
LIS: Lifestyle inflammation scores
DII: Dietary inflammatory index
ADII: Adapted dietary inflammatory index
FBS: Fasting blood sugar
IR: Insulin resistance
CRP: C-reactive protein
HDL-C: High-density lipoprotein-cholesterol
LDL-C: low-density lipoprotein-cholesterol
TG: Triglyceride
BP: Blood pressure
MAPK: Mitogen-activated protein kinase
mTOR: Mammalian target of rapamycin
USDA: United States department of agriculture
FCT: Food composition Table
BMI: Body mass index
WC: Waist circumference
FFQ: Food Frequency Questionnaire
CI: Confidence interval
SD: Standard deviation
IQR: Interquartile range
OR: Odds ratio
