# Supplementary material for: Dietary and lifestyle inflammatory scores and risk of incident diabetes: a prospective cohort among participants of Tehran lipid and glucose study
Source: BMC Public Health. 2021 Jul 2;21:1293. doi: 10.1186/s12889-021-11327-1 (PMC8254228; doi:10.1186/s12889-021-11327-1)
Supplement: Supplementary file 1 — Additional file 1: Table S1. Components of the EDIP, DIS, and LIS indices. [file 12889_2021_11327_MOESM1_ESM.docx]

Table S1. Components of the EDIP, DIS, and LIS indices.

| **EDIP** | |  | **DIS** | |  | **LIS** | |
| --- | --- | --- | --- | --- | --- | --- | --- |
| Components | Weights |  | Components | Weights |  | Components | Weights |
| Processed meat | 165.03 |  | Leafy greens and cruciferous vegetables | − 0.14 |  | Current smoker | 0.50 |
| Red meat | 140.19 |  | Tomatoes | − 0.78 |  | *Physical Activity* |  |
| Organ meat | 144.61 |  | Apples and berries | − 0.65 |  | Moderately physically active | − 0.18 |
| Other fish | 252.45 |  | Deep yellow or orange vegetables and fruit | − 0.57 |  | Heavily physically active | − 0.41 |
| Other vegetables | 136.14 |  | Other fruits and real fruit juices | − 0.16 |  | *BMI* |  |
| Refined grains | 81.21 |  | Other vegetables | − 0.16 |  | Overweight BMI | 0.89 |
| High-energy beverages | 156.85 |  | Legumes | − 0.04 |  | Obese BMI | 1.57 |
| Tomatoes | 167.92 |  | Fish | − 0.08 |  |  |  |
| Tea | − 42.25 |  | Poultry | − 0.45 |  |  |  |
| Coffee | − 83.18 |  | Red and organ meats | 0.02 |  |  |  |
| Dark yellow vegetables | − 165.37 |  | Processed meats | 0.68 |  |  |  |
| Leafy green vegetables | − 190.29 |  | Added sugars | 0.56 |  |  |  |
| Snacks | − 45.08 |  | High-fat dairy | − 0.14 |  |  |  |
| Fruit juice | − 58.95 |  | Low-fat dairy | − 0.12 |  |  |  |
| Pizza | − 1175.21 |  | Coffee and tea | − 0.25 |  |  |  |
|  |  |  | Nuts | − 0.44 |  |  |  |
|  |  |  | Other fats | 0.31 |  |  |  |
|  |  |  | Refined grains and starchy vegetables | 0.72 |  |  |  |

EDIP: empirical dietary inflammatory pattern, DIS: dietary inflammation scores, LIS: lifestyle inflammation scores.
